# Minimum Dietary Diversity and Its Associated Factors Among Pregnant Mothers in Bure District Public Health Facility, North West Ethiopia

**DOI:** 10.1002/fsn3.71331

**Published:** 2025-12-18

**Authors:** Haimanot Degu Mulu, Abraham Degu Mulu

**Affiliations:** ^1^ Department of Public Health Nutrition, College of Medicine and Health Sciences Debre Markos University Debre Markos Ethiopia; ^2^ Students' Clinic Burie Campus Debre Markos University Burie Ethiopia; ^3^ Department of Midwifery, College of Medicine and Health Sciences Bahir Dar University Bahir Dar Ethiopia

## Abstract

Minimum dietary diversity intake during pregnancy increases risks of intrauterine growth restriction, abortion, low birth weight, preterm birth, prenatal, and infant mortality, and morbidity and has long‐lasting health impacts. Dietary diversity during pregnancy promotes the health status of the mother and her fetus. Therefore, this research aimed to address this gap by assessing minimum dietary diversity and associated factors among pregnant women who have given attending antenatal care. To assess the minimum dietary diversity and associated factors among pregnant mothers attending the west Gojjam Zone public health facility, North West Ethiopia, 2025. A facility‐based cross‐sectional study was conducted among 418 pregnant women who attended antenatal care from October 1 to May 12/2025 in west Gojjam Zone public health facilities. The study participants were selected using a systematic sampling technique, and the data were collected through interviews and semi‐structured questionnaires. The data were entered into Epi‐data version 3.1 and exported to Stata version 17 for analysis. Variables with a *p* value of ≤ 0.25 in the bi‐variable logistic regression were used to identify factors associated with maternal dietary diversity. Finally, multivariate logistic regressions were done, and variables that showed *p* values of < 0.05 were considered statistically significant. The magnitude of minimum dietary diversity was 52.5% (95% CI: 47.5, 57.4). Women aged ≥ 34 years (AOR = 3.6, 95% CI: 1.1, 2.4), divorced women (AOR = 0.1, 95% CI: 0.0, 0.2), family size with ≥ 5 member (AOR = 0.4, 95% CI: 0.2, 0.8), able to read and write (AOR = 0.4, 95% CI: 0.2, 0.8), primary school education (AOR = 0.1, 95% CI: 0.0, 0.2), secondary school education (AOR = 0.1, 95% CI: 0.0, 0.2), government employees (AOR = 0.3, 95% CI: 0.1, 0.9), merchants (AOR = 0.1, 95% CI: 0.1, 0.4) and being women head of the household (AOR = 0.2, 95% CI: 0.1, 0.4) those are associated with minimum dietary diversity among pregnant women's. Compared to other earlier investigations, the intake of pregnant mothers with minimum dietary diversity was determined to be minimal. Head of household, family sizes of five or more, women's education, women's residency, and being over 34 years old were the variables that were found to be significantly correlated with minimum dietary diversity. As a result, to encourage maternal dietary diversity, nutrition education, and counseling services should be made available.

## Graphical abstract

Minimum dietary diversity was met by only 52.5% of the 418 pregnant women assessed in Bure district, Ethiopia. Adequate diversity was more common among older women (≥ 34 years), while low education, larger family size, divorce, certain occupations, and female‐headed households were associated with poorer intake. Overall, dietary diversity remains low, underscoring the need for strengthened nutrition education and counseling in antenatal care services.

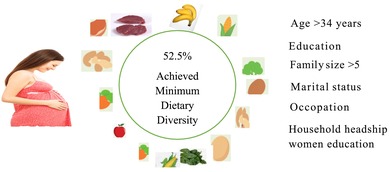

**Abbreviations**
ANC: antenatal care
BMI: body mass index
CI: confidence interval
FANTA: food and nutrition technical assistance
FAO: Food and Agriculture Organization
FQ: food questionnaire
HFIAS: Household Food Insecurity Access Scale
LMP: last manus trial period
MDD: minimum dietary diversity
MUAC: mid‐upper arm circumference
NGO: Non‐Governmental Organization
SIDS: sudden infant death syndrome
UASID: United States Agency for International Development
VIF: Variable Inflation Factor

## 1 Introduction

### 1.1 Background

Poor dietary diversity is a significant public health concern for pregnant women in resource‐poor environments all over the world (Jemal and Awol 2019; Arimond, Torheim, et al. 2008). Dietary diversity is the number of food groups consumed across and within food groups in a given reference period (Arimond, Torheim, et al. 2008). It is a key fundamental aspect of diet quality, which is defined as a diet that provides adequate amounts of selected micronutrients to meet the needs of women of reproductive age and the indicators of nutritional adequacy (Jemal and Awol 2019; Arimond, Torheim, et al. 2008). Minimum dietary intakes in pregnant women are used to reduce maternal mortality and morbidity, and it is also a foundation for the developing fetus's growth and reduced perinatal outcome complications (Engidaye et al. 2019; Sonko 2016). In contrast, suboptimal diets of pregnant women further impact the health of the mother, the developing fetus, and the newborn (Wangalwa et al. 2012).

Minimum dietary diversity assesses the proportion of the feeding practices of pregnant women. If pregnant women feed at least five out of the 10 recommended food groups during the day or night within 24 h, it is considered minimum dietary diversity (Yeneabat et al. 2019). Whereas pregnant women who consume less than five recommended food groups are considered to have poor dietary diversity. The 10 food groups are (1) Grains, white roots and tubers, and plantains, (2) Pulses (beans, peas, and lentils), (3) nuts and seeds, (4) dairy products, (5) meat, poultry, and fish, (6) eggs, (7) dark green leafy vegetables, (8) other fruit rich in vitamin A, (9) other vegetables, and (10) other fruits. A total of 10 food groups were used to classify dietary diversity. Those who consume five or more food groups were classified as having minimum dietary diversity (Food and Agriculture Organization 2021). Proper nutrition is essential for fetal development, reducing the risk of complications such as preterm birth, low birth weight, and gestational disorders (Blumfield et al. 2013). Various socio‐demographic, economic, and cultural factors influence dietary patterns among pregnant women. Education, income level, and food availability significantly impact dietary diversity and nutrition adequacy (Mousa et al. 2019). Additionally, traditional beliefs, cultural practices, and food taboos may restrict the intake of essential nutrients, leading to deficiencies that affect both maternal and neonatal health outcomes (Abu‐Saad and Fraser 2010).

Micronutrient deficiencies, particularly iron, folic acid, and calcium, are common among pregnant women and contribute to adverse health effects (Haider and Bhutta 2017). Healthcare access and nutrition knowledge also play vital roles in shaping dietary behaviors during pregnancy, and women with regular antenatal care visits are more likely to receive dietary counseling, improving their nutritional choices and overall well‐being (Ramakrishnan et al. 2012). Moreover, psychosocial factors such as stress, depression, and food cravings can influence food intake, potentially leading to unhealthy eating behaviors and increased risk of pregnancy complications (Borge et al. 2017). Additionally, low dietary diversity intake during pregnancy raises the risk of low birth weight (LBW), preterm birth, abortion, maternal anemia, intrauterine growth restriction (IUGR), and prenatal and infant mortality, and morbidity and increased risk of chronic disease in the later period of life (Ely and Driscoll 2021; Zerfu et al. 2016). This study aims to address this gap by identifying the major minimum dietary diversity among pregnant women in West Gogjiam Zone, Ethiopia, and analyzing their key predictors. By utilizing a minimum dietary diversity‐based questionnaire and exploratory factor analysis, this research seeks to provide evidence‐based insights that can guide policy recommendations and improve maternal nutrition strategies in Ethiopia. Globally, poor dietary diversity is one of the leading causes of premature death and diseases (Forouzanfar et al. 2015). Lack of dietary diversity among mothers contributes to 7% of the global burden of disease and at least a fifth of maternal mortality and poor maternal outcomes (Desyibelew and Dadi 2019; Zerfu and Biadgilign 2018). One million newborns die on their first day and within their first week of life, linked to increasing rates of maternal anemia, mortality, and adverse outcomes in childbirth (Weldehaweria et al. 2016). In Africa, maternal dietary diversity remains a persistent challenge, with many pregnant women facing a limited variety of food choices due to poverty, food insecurity, and cultural practices (Mirmiran et al. 2021). Studies across Sub‐Saharan Africa have revealed that a majority of pregnant women do not meet the minimum dietary diversity standards, often resulting in micronutrient deficiencies that can lead to maternal anemia and low birth weight (Fawzi et al. 2018). The burden of nutritional deficiency among pregnant women is still high (Lartey 2008). Maternal undernutrition and micronutrient deficiencies can have an impact on the outcomes of pregnancies, raise maternal mortality, and have a negative economic impact (Nuredin et al. 2024; Baer et al. 2005). Macro and micronutrient malnutrition is a major public health concern among pregnant women in developing countries (Ruel et al. 2010), and minimum dietary diversity is affected by urbanization (Asia and Africa) (Zhu and Zhang 2016), income and education levels (Maria Siega‐Riz et al. 2001), and urban middle and high income (Popkin 2014).

In Ethiopia, the maternal mortality ratio is 401 per 100,000 live births, while the neonatal mortality rate is 28 per 1000 live births (Tilahun and Kebede 2021). Furthermore, 8% of women are obese, 24% are anemic, and 22% are underweight (Mengesha et al. 2020). Additionally, 23% and 15% of women of reproductive age in the South Nation of Ethiopia are anemic and thin due to inadequate dietary intake, respectively (Hirvonen and Wolle 2019). In resource‐poor environments across the globe, some dominant plant‐based staple foods and diets lack vegetables, fruits, and animal‐source foods (Arimond, Let, et al. 2008). There is low minimum dietary diversity in Ethiopia among pregnant women (Misganaw et al. 2017). In Ethiopia, the national minimum dietary diversity requirement for pregnant women was only 47% (Hidru et al. 2020). In the Southern Nations, Nationalities, and Peoples Region, 28% of pregnant women were required to have sufficient dietary diversity (Baer et al. 2005). On average, 3.7 of 10 food groups were eaten by pregnant women (Tilahun and Kebede 2021). Dietary diversification through increasing food group consumption in the daily diet is one of the best‐recommended strategies among pregnant women in order to adequately supply the necessary nutrients and lessen the malnutrition of mothers (Biradar 2015). One of the program goals of the second phase National Nutrition Program (NNP II 2016–2020), which the Ethiopian government has developed to address malnutrition, is to improve the nutrition of pregnant and lactating women through thorough and regular dietary assessments and counseling services during the crucial 1000‐day period from conception to the first 2 years of life (Tilahun and Kebede 2021). The primary causes of low dietary diversity in developing nations were found to be environmental, sociodemographic, and economic factors, as well as a lack of nutrition counseling (Lartey 2008; Hidru et al. 2020; Agustina et al. 2023; Savy et al. 2006; Aliwo et al. 2019; Workicho et al. 2016). These factors could vary from setting to setting. Therefore, information on minimum dietary diversity and associated factors of pregnant mothers is urgently needed in order to prioritize, plan, and start intervention programs that will improve the nutrition of mothers and children. However, few studies have documented dietary diversity and the associated factors in the study area. As a result, the purpose of this study was to evaluate maternal dietary diversity and related factors among pregnant women receiving antenatal care (ANC) at the West Gojjam Zone public health facility in North West Ethiopia in 2025.

## 2 Materials and Methods

### 2.1 Study Area

The study was conducted from October 1 to May 12, 2025, at the West Gojjam Zone public health facilities, which are located in Finot Selam town, West Gojjam Zone, which is located in the Amhara region, Ethiopia, 410 km from the capital city of Addis Ababa and 160 km from Bahir Dar, the capital city of Amhara National Regional State. The town has 4 kebeles. It has 2 public health facilities: 4 hospitals and 102 health centers providing basic preventive and medical care in addition to ANC services. In 2025, a total of 500 health professionals and 68 health extension workers are serving the district. According to the district health managers' report, there is an average of 270 women visiting the health facilities within a month for ANC services.

### 2.2 Study Design and Population

This study was a facility‐based cross‐sectional design and was conducted from October 1 to May 12, 2025, in the public health facilities of West Gojjam Zone, Northwest Ethiopia. The study population consisted of randomly selected pregnant women who were attending antenatal care (ANC) services at these facilities during the data collection period. All pregnant women who attended ANC services in the West Gojjam Zone public health facilities during this time were considered the source population. The study included pregnant women who provided informed consent to participate. Pregnant women who were unable to speak or hear, as well as those who were seriously ill during the data collection period, were excluded from the study (Appendix A).

### 2.3 Sample Size Determination and Procedure

The required sample size was determined using a single population proportion formula based on the assumption of a high minimum dietary diversity of 55.2% in Gondar, Ethiopia (Workneh et al. 2021). With a margin of error of 5% and a 95% confidence interval, and adding 10% for the non‐response rate, therefore, the minimum sample size will be calculated as follows:

$$n=\frac{{\left(\mathrm{Za}/2\right)}^{2}*p\left(1-p\right)}{d^{2}}$$

$$n=\frac{{\left(1.96\right)}^{2}*0.552\left(1-0.448\right)}{{\left(0.05\right)}^{2}}=\frac{3.84*0.2472}{0.0025}=380$$

Here: *n* = sample size.

Proportion of minimum dietary diversity 0.552 (55.2%) CI = Confidence interval with 95%.

Za/2 = Critical value at 95% confidence level of certainty is (1.96), the margin of error to be tolerated and taken as 5%. Considering a 10% contingency for missing data, 0.1*380 = 38 = 38 + 380 = **418**.

### 2.4 Variables of the Study

#### 2.4.1 Dependent Variable

Minimum dietary diversity (Good or Poor).

#### 2.4.2 Independent Variable

Socio‐demographic factors: Age, marital status, religion, residence, education, occupation, head of household, family size, monthly income, nutrition awareness, husband support, and nutritional status.

Nutrition Awareness and Counseling factors: Nutrition awareness and Nutrition Counseling.

Economic and food security factors: Household income, food security status, bank account ownership, mobile phone ownership, radio ownership, and access to a home garden.

Environmental and health factors: Source of drinking water, presence of a latrine at home, and MUAC.

### 2.5 Operational Definition

Minimum dietary diversity: A pregnant woman consumed at least 5 out of 10 recommended food groups over the past 24 h (Prista et al. 2003).

Inadequate dietary diversity: refers to the consumption of a limited variety of food groups (Gustafsson et al. 2013).

Poor dietary diversity is considered when pregnant women attain a high dietary diversity (< 5 food groups) (Prista et al. 2003).

Nutritional status: MUAC Circumference will be measured using non‐stretchable tape, and a cut‐off point for MUAC > 22 cm normal, and < 23 cm will be considered underweight. MUAC has been recommended as it has been found to be a potential indicator of nutritional status during pregnancy (Desta et al. 2019).

### 2.6 Data Collection Tool and Procedure

#### 2.6.1 Data Collection Tool

The data were gathered in face‐to‐face interviews using pretested, organized, and semi‐structured questionnaires that were derived from various types of literature (Jemal and Awol 2019; Gómez et al. 2024; Shenka et al. 2018). The data were gathered by Midwife Nurse Professionals. The questionnaire has two parts. Socio‐demographic characteristics, environmental and health‐related characteristics, economic and food security characteristics, and Nutrition Awareness and Counseling characteristics are included in the first component. The second component consisted of dietary‐related data questionnaires that were taken from the Food and Agriculture Organization of the United Nations (FAO) 2016 (Shenka et al. 2018). The dietary diversity questionnaire divides foods into 10 categories according to their nutrients: (1) grains, white root, tubers, and plantains; (2) pulses (beans, peas, and lentils); (3) nuts and seeds; (4) dairy; (5) meat and fish (poultry and fish); (6) eggs; (7) dark green leafy vegetables; (8) vitamin A‐rich fruits and vegetables; (9) other vegetables; and (10) other fruits. It was assessed by using a 24‐h open dietary recall method; one point was given to each food group consumed over the past 24 h before the survey period. The participants were asked about all food and beverages consumed during the day and night, including any snacks in the past 24 h, and the interviewer will probe for any food types forgotten by participants. Each food or beverage that the respondent mentions was circled and underlined on a predefined list. The foods not included on the predefined list were classified by the principal investigator into an existing predefined food group or recorded in a separate place on the questionnaire and coded and organized later into one of the predefined food groups (Shenka et al. 2018) (Appendixes B and C).

The nutritional status of pregnant women was evaluated by measuring their mid‐upper arm circumference (MUAC), which is the distance between the olecranon process and acromion process as measured by non‐stretchable tape to the nearest 0.1 cm. Nutritional status was defined as MUAC < 22 cm was considered nutritional, while 22 cm or more was considered to be normal nutritional status (Belete et al. 2016).

#### 2.6.2 Data Collection Procedure

The data was collected from four public health facilities that provide ANC services (one primary hospital and three Health Centers). A systematic random sampling technique was used to select study participants. First, the total sample size was proportionally allocated to each public health facility based on the ANC flow in the previous month. Then the sampling interval (*K*) was determined by dividing the number of mothers who gave ANC in a month by the required sample. The total ANC in the previous month at Aserade Zewudie Primary Hospital, Bure Health Center, Tataya Health Center, and Derekohuwa Health Center in four public health facilities was 869, then divided by a sample size of 481, *K* = 2.08. The first mother will be chosen by the lottery method from one of the two ANCs on the first day of delivery in each health facility. One participant was selected from every other ANC until the needed sample size was attained in each facility. The data for this study were collected within a day by interviewing the mothers when they are in comfort and stable.

### 2.7 Data Quality Assurance

The pre‐test was conducted in 5% of the total sample size of Bure Yohannes Hospital outside of the study area on pregnant women attending ANC in the Bure district public health facility, and the questionnaire was assessed for its content, length, and word selection. This helps in changing the questionnaires by including missed meals or excluding foods that are not relevant to the study areas. One MSc midwife and two BSc midwives were given training to the data collectors and the supervisor, who will train for 2 days before the data collection on the whole data collection procedure. The procedure manual for the data collection was prepared and distributed to the data collectors and the supervisor. During the data collection, the principal investigator and the supervisor reviewed the filled questionnaires daily. Before the data entry, each questionnaire was given a unique code by the principal investigator.

### 2.8 Data Analysis Technique

The completed questionnaires have been cleaned and coded, and the data were entered into Epidata 3.1 software and exported to Stata 17 for analysis. A minimum dietary diversity score (MDDS) will be dichotomized as meeting minimum dietary diversity for pregnant women who consumed 5 or above out of 10 food groups coded as 0, and not meeting minimum dietary diversity for those who consumed fewer than 5 out of 10 food groups in the past 24 h coded as 1 (Food and Agriculture Organization 2021). Descriptive analysis will be done to calculate the mean, frequency, and percentage distributions for the variables. Logistic regression was done to identify variables associated with minimum dietary diversity. The model fitness was checked by using the Hosmer and Lemeshow statistic test. Bivariate logistic regression with a *p* < 0.25 was done to identify the variables associated with minimum dietary diversity and independent variables, and the variables with a *p* < 0.05 were considered for multivariable logistic regression to control all possible confounders and to determine the strength of association between minimum dietary diversity and each explanatory variable. The strength of association was measured with an adjusted odds ratio (AOR) with a 95% CI. Finally, the variables with a *p* < 0.05 were considered statistically significant.

### 2.9 Ethical Consideration

Ethical clearance was obtained from the Institutional Review Board of Debre Markos University College of Medicine and Health Sciences, and official permission was obtained from West Gojjam Zone public health facilities. Information was addressed to participants, and we reassured them that anyone who is not willing to take part in the study has the full right to refuse, and those involved can also leave the study if they are not comfortable. After getting permission from each participant, the sample was collected from the selected participants. In this study, the interview was conducted; information provided by the participants has been kept confidential.

## 3 Results

### 3.1 Socio‐Demographic Characteristics of Pregnant Women

A total of 408/418 pregnant women participated in this study with a response rate of 98%. Most of the participants were married (194, 47.6%). The majority (36.8%) of the respondents were not educated. We were Orthodox religious followers, and the majority of participants (51.7%) were married. Around 15.2% of pregnant women attended primary school according to their educational status. Regarding the occupation, 33.8% of pregnant women were housewives, and more than half of the respondents were rural residents. About 53.4% of participants had a family size of ≥ 5, and 53.7% of pregnant women's household heads were husbands (Table 1).

**TABLE 1:** Socio‐demographic characteristics of pregnant women at the West Gojjam Zone public health facilities, North West Ethiopia, 2025 (*N* = 408).

Variable	Category	Frequency	Percent
Age of women	< 25	69	16.9
	26–34	201	49.3
	≥ 34	138	33.8
Marital status	Single	66	16.2
	Married	194	47.6
	Widowed	114	27.9
	Divorced	34	8.3
Women religion	Orthodox	211	51.7
	Muslim	94	23.0
	Protestant	73	17.9
	Catholic	30	7.4
Residence	Urban	151	37.0
	Rural	257	63.0
Women education	Unable to read and write	150	36.8
	Able to read and write	117	28.7
	Primary (1–8)	62	15.2
	Secondary (9–12)	40	9.8
	College and above	39	9.6
Women occupational	Housewife	138	33.8
	Government employee	82	20.0
	NGO employee	46	11.3
	Merchant	31	7.6
	Daily labor	111	27.2
Head of household	Husband	219	53.7
	Wife	156	38.2
	Both	33	8.1
Family size	< 5	190	46.6
	≥ 5	218	53.4

### 3.2 Assessment of Economic, Environmental, and Food Security Characteristics of Pregnant Women

Among the respondents, nearly half of the pregnant women were receiving health information from the radio (49%). In terms of financial access, 42.4% of participants reported having a bank account. Regarding water sources, 16.9% of respondents used tap water, 19.4% used spring water, and the majority (63.7%) relied on well water (Table 2).

**TABLE 2:** Assessment of economic, environmental, and food security characteristics of pregnant women at the West Gojjam Zone public health facilities, North West Ethiopia, 2025 (*N* = 408).

Variable	Category	Frequency	Percent
Income	< 1500	168	41.2
	≥ 1500	240	58.8
Shopping	Wife	256	62.8
	Husband	78	19.1
	Others	74	18.2
Bank account	Yes	173	42.4
	No	235	57.6
Mobile	Yes	211	51.7
	No	197	48.3
Radio	Yes	200	49.0
	No	208	51.0
Garden	Yes	154	37.8
	No	254	62.3
Latrine	Yes	105	25.7
	No	303	74.3
Water source	Tab water	69	16.9
	Spring water	79	19.4
	Well water	260	63.7
Nutritional status	< 22	19	4.7
	≥ 22	389	95.3
Nutrition awareness	Yes	270	66.2
	No	138	33.8

### 3.3 Minimum Dietary Diversity Consumption Patterns of Pregnant Women

**FIGURE 1:**
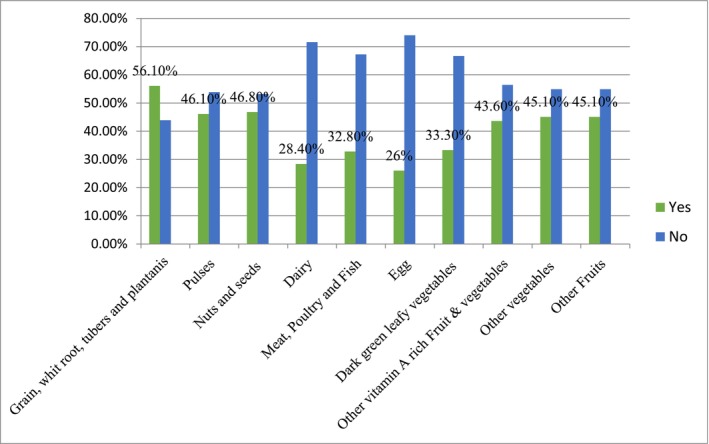
Minimum dietary diversity consumption patterns of pregnant women.

Regarding the food groups consumed by pregnant women in the previous 24 h, about 56.1% consumed starchy staple food groups, 46.1% consumed pulses, 46.8% consumed nuts and seeds, and 43.6% consumed other vitamin–A–rich fruits and vegetables, while the least consumed food groups were dairy (28.4%) and meat and fish (32.8%) (Figure 1; Table 3).

**TABLE 3:** Food groups' consumption patterns of pregnant women at West Gojjam Zone public health facilities, North West Ethiopia, 2025 (*N* = 408).

Food group consumption in the previous 24 h	Category	Frequency	Percent
Grain, white root, tubers, and plantains	Yes	229	56.1
	No	179	43.9
Pulses	Yes	188	46.1
	No	220	53.9
Nuts and seeds	Yes	191	46.8
	No	217	53.2
Dairy	Yes	116	28.4
	No	292	71.6
Meat, Poultry, and Fish	Yes	134	32.8
	No	274	67.2
Egg	Yes	106	26.0
	No	302	74.0
Dark green leafy vegetables	Yes	136	33.3
	No	272	66.7
Other vitamin A‐rich Fruits and vegetables	Yes	178	43.6
	No	230	56.4
Other vegetables	Yes	184	45.1
	No	224	54.9
Other fruits	Yes	184	45.1
	No	224	54.9

### 3.4 Prevalence of Minimum Dietary Diversity

**FIGURE 2:**
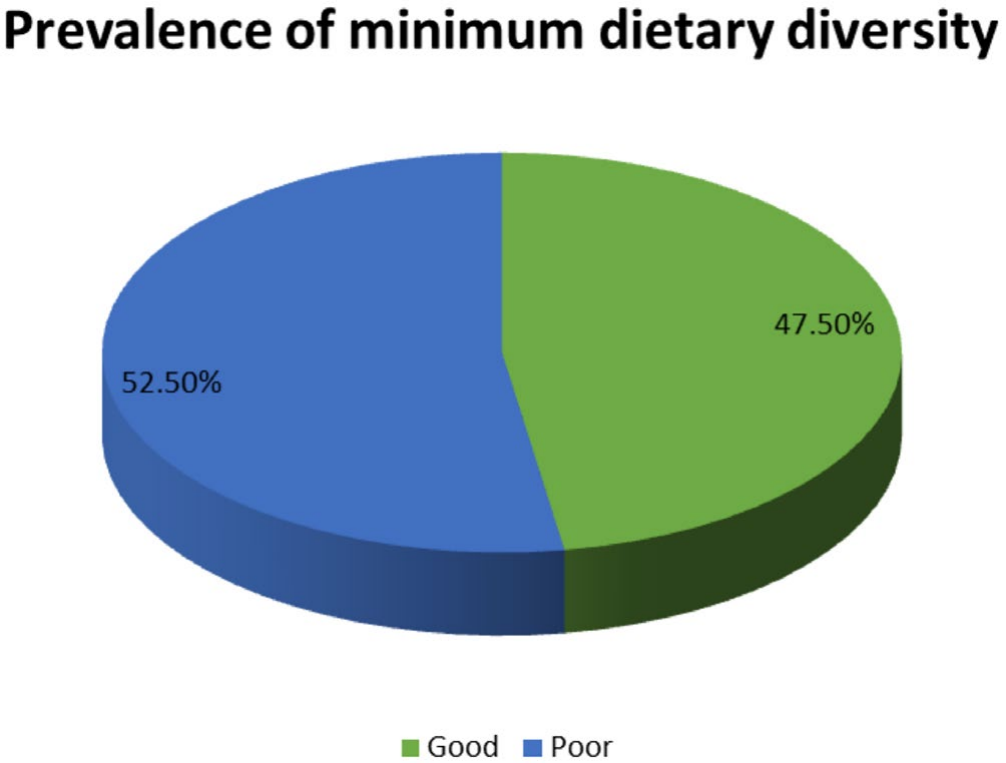
Prevalence of minimum dietary diversity among pregnant women.

The overall magnitude of minimum dietary diversity in pregnant women was 52.5% (95% CI: 47.5–57.4) (Figure 2).

### 3.5 Factors Associated With Minimum Dietary Diversity in Pregnant Women

In bi‐variable logistic regression analysis, the variables: Age of women between 26 and 34, marital status, family size, residence in rural areas, women's education, women's occupation being employee, nutrition awareness, and head of household being wife were significantly associated (*p* < 0.25) with the minimum dietary diversity score. Subsequently, in a multivariable binary logistic regression analysis, the age of women, marital status, residence, women's education, women's occupation, maternal nutrition awareness, and head of household were significantly associated with the minimum dietary diversity score, at a *p*‐value of < 0.05. Pregnant women in the age group of 26–34 years had significantly lower odds of inadequate dietary diversity (AOR: 0.2, 95% CI: 01, 0.5), which means 80% less likely to have poor diversity compared to women < 25 years. Married women had significantly higher dietary diversity (AOR = 5.0, 95% CI: 2.0, 12.4) 5 times more likely to have adequate dietary diversity compared to single women. Widowed women had significantly higher dietary diversity (AOR = 4.9, 95% CI: 1.9, 12.9) 4.9 times more likely to have adequate dietary diversity compared to single women. Divorced women had significantly higher dietary diversity (AOR = 5.4, 95% CI: 1.5, 18.5) 5.4 times more likely to have adequate dietary diversity compared to single women. Rural‐residing women were significantly less likely to have adequate dietary diversity (AOR =0.3, 95% CI: 0.2, 0.6) and had a 70% higher likelihood of poor diversity compared to urban women. The pregnant women who received nutrition information during pregnancy were 70% less likely to (AOR = 0.3, 95% CI: 0.2, 0.5) meet the minimum dietary diversity score than those who didn't get nutrition information (Table 4).

**TABLE 4:** Multivariable logistic regression of factors associated with minimum dietary diversity in pregnant women at the West Gojjam Zone public health facilities, North West Ethiopia, 2025 (*N* = 408).

Variables	Category	Dietary diversity		COR, 95% CI	AOR, 95% CI
		Good	Poor		
Age of women	< 25	17	25	1	1
	26–34	101	141	1.1 (0.5, 2.1)	1.0 (0.3, 2.9)
	≥ 34	76	48	2.3 (1.1, 4.8)*	3.6 (1.1, 2.4)**
Marital status	Single	16	50	1	1
	Married	101	93	2.8 (1.3, 5.8)*	2.3 (0.7, 7.2)
	Widowed	63	51	1.9 (0.9, 4.1)	2.7 (0.9, 8.4)
	Divorced	14	20	0.2 (0.1, 0.5)*	0.1 (0.0, 0.2)**
Family size	< 5	107	93	1	1
	≥ 5	87	131	0.5 (0.3, 0.8)*	0.4 (0.2, 0.8)**
Residence	Urban	95	56	1	1
	Rural	99	158	0.4 (0.2, 0.6)*	0.3 (0.2, 0.6)**
Women education	Unable to read or write	80	70	1	1
	Able to read & write	41	76	0.2 (0.1, 0.3)*	0.4 (0.2, 0.8)**
	Primary (1–8)	32	30	0.1 (0.1, 0.2)*	0.1 (0.0, 0.2)**
	Secondary (9–12)	10	30	0.1 (0.1, 0.3)*	0.1 (0.0, 0.2)**
	College & above	31	8	2.6 (0.8, 8.1)	2.3 (0.5, 10.6)
Women occupation	Housewife	89	49	1	1
	Govt employee	35	47	0.7 (0.4, 1.3)	0.3 (0.1, 0.9)**
	NGO employee	15	31	0.5 (0.3, 0.9)*	0.1 (0.0–0.4)**
	Merchant	6	25	0.6 (0.3, 1.0)*	0.1 (0.1, 0.4)**
	Daily labor	49	62	0.2 (0.1, 0.4)*	0.0 (0.0, 0.1)**
Head of household	Both	41	33	1	1
	Wife	47	124	0.3 (0.2, 0.5)*	0.2 (0.1, 0.4)**
	Husband	106	57	1.5 (0.9, 2.6)	2.1 (0.9, 5.3)

* Significant at *p* < 0.25 in unadjusted logistic regression analysis.

** Significant at *p* < 0.05 in adjusted logistic regression analysis.

## 4 Discussion

The current study found that 52.5% of pregnant women had consumed fewer than five food groups in the previous 24 h, compared to that of the (AOR = 0.4, 95% CI: 0.2, 0.8) who had consumed more than five food groups. It is lower than the study findings from Burkina Faso (64.4%) (Savy et al. 2006), Indonesia (57.9%) (Diana et al. 2019), Addis Ababa (60.9%) (Tefera et al. 2020), Hosanna town of Ethiopia (75.8%) (Delil et al. 2018), and Alamata (61.2%) (Jemal and Awol 2019). The discrepancy might be due to differences in the study settings since the current study was conducted among rural residents with little access to nutrition education and health services. Moreover, respondents in this study had a low level of education. Their literacy level might have been a barrier to accessing information related to good nutrition practices (Demilew et al. 2020). The finding is higher than similar Ethiopian studies in Dessie town (45.2%) (Diddana 2019), west Gojam (19.9%) (Demilew et al. 2020), Gonder town (40.1%) (Yenealem and Niberet 2019), Misha wereda (29.5%) (Abute et al. 2020), Shahemene town (25.4%) (Desta et al. 2019) and Public Health Institution in Mizan‐Aman Town (25.1%) (Girma Tilahun et al. 2021). The possible reasons might be demographic and cultural differences, as previous studies were conducted in areas where the habit of cultivating and consuming vegetables/fruits was low (Demissie et al. 2009), and the cut‐off point difference.

According to this study, the minimum dietary diversity of pregnant women in the age group of > 34 years was 33.8% more likely to have poor minimum dietary diversity scores than compared to those in the age group of < 34 years old. The possible explanation for this could be that women who are older contribute to the higher risk of insufficient minimum dietary diversity in pregnant women over 34 years of age. Older women may place the nutritional needs of their family members above their own, which may lead to less varied diets. In addition, they may be more likely to adhere to conventional eating habits, which limit the range of foods they can consume during pregnancy. This may also be due to a lack of exposure to nutrition education programs, which often target younger or first‐time mothers. Because of the generational differences in nutrition knowledge and attitudes, studies have shown that maternal age may influence minimum dietary habits (Kavle and Landry 2018; Hanley‐Cook et al. 2024). This is supported by (Sinclair 2021; al GbSe 2017; Tetteh 2020).

We found that participants who had secondary and lower‐level education had a poor habit of dietary diversity within their diet as compared with those who were in college and above. This might be due to poor knowledge about the types of food groups that may be consumed within 24 h to achieve their minimum dietary diversity level. Women with higher education tend to include a variety of food groups and good dietary practices in their diet (Murakami et al. 2009). Less education may be linked with poor food choices and preparation due to a lack of knowledge and resources for their dietary diversity (Mazur et al. 2003). The study was supported by (Darmon and Drewnowski 2015, 2008; Spronk et al. 2014; Stringhini et al. 2017). Pregnant women living in households with more than five members had higher odds of inadequate minimum dietary diversity. Larger family size often leads to increased competition for limited household food resources, resulting in reduced individual dietary quantity and diversity, especially for vulnerable members like pregnant women (Leser 2013). This finding superseded that (Workicho et al. 2016).

The study suggested that government employees, NGO workers, merchants, and daily laborers may still face poor dietary diversity for some reasons because women in war‐affected zones still experience poor dietary diversity due to multiple conflict‐related challenges. First, war disrupts food supply chains, limiting the availability of diverse and nutritious foods in local markets. Second, inflation and unstable income reduce the purchasing power of even salaried workers like government and NGO employees. Third, insecurity and displacement restrict women's access to markets and food sources, especially in rural areas. Finally, the interruption of health and nutrition services during conflict leaves pregnant women without proper dietary guidance and support (Pérez‐Escamilla et al. 2017; Cooper 2018; World Health Organization 2021). Our study supported that (FAO WDFSC 2020; Corral Rodas et al. 2020). Furthermore, women who were heads of households had greater odds of attaining adequate dietary diversity compared to those whose husbands were the heads of households in Bangladesh (Nguyen et al. 2017) and Tanzania (Ochieng et al. 2017) The possible explanation for this could be that those women who had household heads may have more control over household resources and decision‐making, including food purchasing and preparation, which can positively influence their dietary diversity. When women have the autonomy to make independent decisions regarding food, they are more likely to prioritize diverse and nutritious diets, especially during pregnancy. This study supported that in Bangladesh (Nguyen et al. 2017).

## 5 Strengths and Limitations

This study's strengths include the use of a standardized tool to assess minimum dietary diversity, a representative sample size, and the consideration of a wide range of variables, which together help fill an important knowledge gap regarding maternal nutrition in the study area. However, as a cross‐sectional design, the study cannot establish cause‐and‐effect relationships. Additionally, seasonal variation in food availability was not considered, and the study did not assess nutrient levels in the foods consumed. Other limitations include geographic, cultural, and occupational constraints that may affect generalizability, reliance on self‐reported data, and potential recall bias in reporting minimum dietary intake within the past 24 h.

## 6 Conclusion and Recommendations

### 6.1 Conclusion

This study revealed that the overall level of adequate dietary diversity among pregnant mothers in the Bure district was low. Factors such as maternal age over 34, rural residence, larger family size (≥ 5 members), lower educational status, and household headship were significantly associated with dietary diversity. These findings highlight the need to strengthen nutrition education and counseling services during antenatal care to promote better dietary practices among pregnant women. Special attention should be given to older and rural‐dwelling women, as they are at higher risk of inadequate dietary intake. Pregnant mothers should be encouraged to diversify their diets by including more animal‐source foods and various types of fruits. Additionally, further research is recommended to explore in greater depth the impact of maternal age and rural residence on poor dietary diversity during pregnancy.

### 6.2 Recommendations

To improve maternal nutrition, targeted nutrition education should be delivered during antenatal care to inform pregnant women about the importance of dietary diversity and nutrient‐rich foods. Economic support mechanisms such as food subsidies, home gardening initiatives, and cash transfers should be implemented to enhance access to diverse foods for low‐income households. Community awareness must be strengthened through channels like radio, mobile messaging, and community health workers to challenge food taboos and encourage healthy eating habits. Engaging husbands and other household members in nutrition education can promote shared responsibility and ensure pregnant women receive adequate food support. Regular monitoring of pregnant women's nutritional status, including dietary intake and MUAC, is essential to detect and address deficiencies early. Finally, all interventions should be aligned with Ethiopia's National Nutrition Program to ensure their sustainability and broader impact on maternal and child health.

## Author Contributions

**Haimanot Degu Mulu:** conceptualization, data curation, formal analysis, funding acquisition, investigation, methodology, project administration, resources, software, validation, visualization, and writing – original draft. **Abraham Degu Mulu:** conceptualization, data curation, formal analysis, methodology, project administration, supervision, validation, visualization, and writing – review and editing.

## Funding

The authors have nothing to report.

## Ethics Statement

This study involves human data, and all research methods and procedures were performed following the Declaration of Helsinki after the ethical clearance was obtained from the ethical review board of Debre Markos University. A formal permission letter was written to the West Gojjam Zone Health Department. After that, a permission letter was obtained from each office director to cascade the research.

## Consent

After explaining the purpose of the study, the data collectors obtained written consent from each study participant. The participants were informed that participation was on a voluntary basis and they could withdraw from the study at any time if they were not comfortable with the questionnaire. The personal identifier was not included so that a participant's confidentiality was assured.

## Conflicts of Interest

The authors declare no conflicts of interest.

## Data Availability

The data that support the findings of this study are available on request from the corresponding author. The data are not publicly available due to privacy or ethical restrictions.

## Appendix A Information and Consent Sheet

Debre Markos University College of Medicine and Health Sciences Department of Public Health.

Hello, my name is _____________ I am working as a data collector with Haimanot Degu, who is completing his Master's Degree in Public Health at Debre Markos University. He is conducting a study on “Minimum Dietary diversity and Associated Factors among pregnant mothers in Bure district public health facility, North West Ethiopia, 2025”. This study is a part of the requirements for the fulfillment of the MSc program he is enrolled in. We will ask you questions about your health and household. Your participation in this study is voluntary, and you may stop the interview at any time when you get bored. The interview will take just 30 min. The data we collect from you is entirely confidential, and your answers will not be given to anyone; rather, they will be used only for this research. Your name will not be identified in the report. We would like to have complete data, but you do not have to answer questions you do not want to. However, I hope that you will participate in this study since your views are important. Finally, I would like to assure you that no risk will occur by participating in the study. For detailed information, you can contact the investigator through cell phone at +251913360266 and e‐mail haimidegu@gmail.com.

- If you have any questions about the survey, I am ready to respond. Do you have any questions?
- Please let me know if anything I have stated is not clear, and I will be happy to explain it further to ensure you understand.

I have been informed about the purpose and use of this particular research. After all this, I understood and:

1. I agree to participate in this research voluntarily
2. I didn't agree to participate in this research

Interviewer's name Signature Date → Signature → Date →

**Thank you in advance for your cooperation in the study**

## Appendix B English Version Questionnaire

Name of the interviewer __________ Date of interview

___________ Date of visit _________ CODE ______ DD |MM |YYYY.

**Table:**

**I. Assessment for Socio‐Demographic Information Questions**			
**No**.	**Questions**	**Responses**	**Code**
101	What is your age?	_______?	
102	What is your husband's age?	_____________	
103	What is your marital status?	0 Single 1 Married 2 Divorced 3 Widowed	
104	What is your religion?	0 Orthodox 1 Muslim 2 Protestant 3 Catholic	
105	Where do you live?	0 Urban 1 Rural	
106	What is your educational status?	Unable to read and write 0 Able to read and write 1 Primary school (1–8) 2 Secondary school (9–12) 3 College and above	
107	What is your husband's educational status?	Unable to read and write 0 Read and write 1 Primary (1–8) 2 Secondary (9–12) 3 College and above	
108	What is your occupation?	0 Housewife 1 Government employee 2 NGO employee 3 Merchant 4 Daily labor	
109	Who is the head of the household	0 Husband 1 Wife 2 Both	
110	What is your Family size?	________?	
**II. Assessment of economic and food security factors**			
111	What is your household income?	____________?	
112	Who is responsible for shopping in your household?	0 Wife 1 Husband 2 Other	
113	Do you have a bank account?	0 Yes 1 No	
114	Do you have a mobile phone?	0 Yes 1 No	
115	Do you have a radio at home?	0 Yes 1 No	
116	Do you have a garden at home?	0 Yes 1 No	
**III. Assessment of Environmental and Health Factors**			
117	Do you have a latrine at home?	0 Yes 1 No	
118	What is your main source of water?	0 Tap 1 Spring 2 Well	
119	What is your mid‐upper arm circumference (MUAC) measurement?	________?	
**IV. Assessment Dietary‐Related Information**			
120	Did you consume grains, white roots, tubers, or plantains in the last 24 h?	0 Yes 1 No	
121	Did you consume pulses (beans, peas, lentils) in the last 24 h?	0 Yes 1 No	
122	Did you consume nuts or seeds in the last 24 h?	0 Yes 1 No	
123	Did you consume dairy products in the last 24 h?	0 Yes 1 No	
124	Did you consume meat or fish in the last 24 h?	0 Yes 1 No	
125	Did you consume eggs in the last 24 h?	0 Yes 1 No	
126	Did you consume dark green leafy vegetables in the last 24 h?	0 Yes 1 No	
127	Did you consume vitamin A‐rich fruits and vegetables in the last 24 h?	0 Yes 1 No	
128	Did you consume other vegetables in the last 24 h?	0 Yes 1 No	
129	Did you consume other fruits in the last 24 h?	0 Yes 1 No	
130	Have you received any information about the importance of dietary diversity during pregnancy?	0 Yes 1 No	

## Appendix C Amharic Version Questionnaire

በደብረማርቆስ ዩኒርሲቲ የድህረ ምረቃ ፕሮገራም በህብረተሰብ ጤና ትምህርት ክፍል የሁለተኛ ዲግሪ ተማሪዎች ጥናት ለመስራት የተዘጋጀ ነው፡፡

የጥናቱ አላማ መግለጫና የስምምነት ቅፅ

ጤና ይስጥልኝ! ስሜ ይባላል፡፡ እኔ ከሀይማኖት ደጉ ጋር በመረጃ ሰብሳቢነት እየሰራሁ ሲሆን ይህ ጥናት በደብረማርቆስ ዩኒርሲቲ የድህረ ምረቃ ፕሮገራም በህብረተሰብ ጤና ትምህርት ክፍል የሁለተኛ ዲግሪ ተማሪዎች ጥናት ለመስራት የተዘጋጀ መስክ ለመመረቅ ከሚያስፈልጎት መስፈርቶች አንዱና ዋነኛው ነው፡፡ ጥናቱን የሚያካሂደው ቡሬ ከተማ ውስጥ ወደሚገኙ የመንግስት ጤና ተቋማት የሚመጡ ነፍሰጡር እናቶችን የምግብ ስብጥር እና የስርዓተ‐ምግብ ሁኔታን ለማጥናት ሲሆን ከስርዓተ‐ ምግብ ሁኔታቸው ጋር ተያያዥነት ያላቸውን ማህበራዊ ሁኔታዎች እና ሌሎች ምክንያቶችን ለይቶ ለማወቅ ነው፡፡ የማህበራዊ እና ሌሎች ሁኔታዎችን በተመለከተ ጥያቄዎች እንጠይቀወታለን:: በዚህ መጠይቅ ላይ የርሶ ተሳትፎ በሙሉ ፈቃደኝነት ላይ የተመሰረተ ሲሆን ድብርት ባጋጠመዎት በየትኛውም ጊዜ መጠይቁን የማቋረጥ ሙሉ መብት አለዎት፡፡ ይህ መጠይቅ 30 ያህል ደቂቃዎችን ይወስዳል::

በዚህ ጥናት ከእርሶ የሚገኘውን መረጃ ሚስጥራዊነቱን መጠበቅ ዋናው ስራችን ሲሆን ከርሶ የተሰበሰቡት መረጃዎች ለማንም ሰው የማይሰጡና ለጥናቱ አላማ ብቻ የሚውሉ ይሆናሉ፡፡ ስመዎም በሪፖርቱ ውስጥ አይካተትም፡፡ ከርሶ የተሟላ መረጃ እንድኖረን እንፈልጋለን፤ ነገር ግን ለመመለስ የማይፈልጉት ጥያቄ ካለ አለመመለስ የሚችሉ ሲሆን በጥናቱ እንደሚሳተፉ ተስፋ እናደርጋለን፤ ምክንያቱም የርሶ እይታዎች ጠቃሚ ናቸውና፡፡ በመጨረሻም በዚህ ጥናት በመሳተፈዎ ምንም አይነት ጉዳት አይደርስበዎትም፡፡ ለበለጠ መረጃ በስልክ ቁጥር +251913360266 ወይም በኢሜል አድራሻ haimidegu@gmail.com መጠቀም ይችላሉ፡፡

ጥናቱን በተመለከተ የትኛውም አይነት ጥያቄ ካለዎት ጥያቄዎትን ለመመለስ ዝግጁ ነኝ፡፡ ጥያቄ አለዎት?

እስካሁን ከገለፅኩለዎት ግልፅ ያልሆነለዎት ነገር ካለ እባከዎ ልዎቀው፣ በትክክል የተረዱኝ መሆኑን ለማረጋገጥ ከዚህ የበለጠ ማብራሪያ ከፈለጉ ባብራራለዎት ደስተኛ ነኝ፡፡ የጥናቱ አላማና ጥቅም ተረድቸዋለሁ በመሆኑም ማንኛውም የምሰጠው መረጃ ለዚህ ጥናት ብቻ እንደሚውል እና ማንነቴ የማይገለጽ መሆኑን ስለተረዳው በዚህ ጥናት ላይ ለመሳተፍ፡

የጠያቂው/ዋ ስም_____________ፊርማ___________ቀን_________በፍቃደኝነት ስለሚያደርጉት አስተዋኦ በቅድሚያ እናመሰግናለን::

በፍቃደኝነት ስለሚያደርጉት አስተዋኦ በቅድሚያ እናመሰግናለን::

የጤና ተቋሙ ስም __________________________________________________

የጉብኝት ቀን ________ → |___________ → |_________ → መለያ ቁጥር________ → ቀን | ወር | ዓ.ም0

**Table:**

**ክፍል 1: የእናቶችን ማህበራዊ እና አካባቢያዊ ሁኔታዎችን የተመለከቱ ጥያቄዎች**			
**ተ.ቁ**	**ጥያቄዎች**	**አማራጮች/መልስ መስጫ**	**ኮድ**
101	እድሜዎ ስንት ነው?	________________?	
102	የባለቤትዎ እድሜዎ ስንት ነው?	___________?	
103	የትዳር ሁኔታዎ ምንድነው?	0 ያላገቡ 1 ያገቡ/ባለትዳር 2 የፈቱ 3 ባሏ የሞተባት/ሚስቱ የሞተችበት	
104	ሀይማኖትዎ ምንድን ነው?	0 ኦርቶዶክስ 1 ሙስሊም 2 ፕሮቴስታንት 3 ካቶሊክ	
105	የመኖርያ አካባቢዎ የት ነው?	0 ከተማ 1 ገጠር	
106	የትምህርት ደረጃዎ የትኛው ነው?	0 ማበብ እና መጻፍ የማልችል 1 ማበብ እና መጻፍ የምችል 2 የመጀመሪያ ደረጃ ትም/ቷን ያጠናቀቀች(1‐8) 3 የ2ኛ ደረጃ ትም/ቷን ያጥናቀቀች (9‐12) 4 የኮሌጅ ድፕሎማና ከዛ በላይ	
107	የባለቤትዎ የትምህርት ደረጃዎ የትኛው ነው?	0 ማበብ እና መጻፍ የማይችል 1 ማበብ እና መጻፍ የሚችል 2 የመጀመሪያ ደረጃ ትም/ያጠናቀቀ(1‐8) 3 የ2ኛ ደረጃ ትም/ት ያጥናቀቀ (9‐12) 4 የኮሌጅ ድፕሎማና ከዛ በላይ	
108	የቤተሰበዎ ሃላፊ ማነው?	0 ባል 1 ሚስት 2 ሁለቱም	
109	ስራዎ ምንድን ነው?	0 የቤት እመቤት 1 የቀን ሰራተኛ 2 የመንግስት ሰራተኛ 3 መንግስታዊ ያልሆነ ድርጅት ሰራተኛ 4 ነጋዴ	
110	የቤተሰብዎት ብዛት በቁጥር ስንት የሆናሉ	______________?	
**ክፍል II የኢኮኖሚ እና የምግብ ዋስትና ሁኔታዎች ግምገማ**			
111	የቤተሰብዎ የወር ገቢ ስንት ነው	_______________?	
112	የቤት አስቤዛ የሚገዛው ማነው?	0 ሚስት 1 ባል 2 ሌላ	
113	የባንክ አካውንት አለዎት?	0 አዎ 1 የለም	
114	የሞባይል ስልክ አለዎት?	0 አዎ 1 የለም	
115	ሬዲዮ አለዎት?	0 አዎ 1 የለም	
116	ቤት ውስጥ የአትክልት ቦታ አለዎት?	0 አዎ 1 የለም	
**ክፍል III. የአካባቢ እና የጤና ሁኔታዎች ግምገማ**			
117	በቤትዎ በብዛት የሚጠቀሙት የውሃ ምንጭ ምንድነው?	0 ታፕ 1 የምንጭ ዉሃ 2 የጉድጓድ ውሃ	
118	ቤት ውስጥ ሽንት ቤት አለ?	0 አዎ 1 የለም	
119	የመሃል የላይኛው የክንድ ዙሪያ (MUAC) ልኬቱ ስንት ነው	______________?	
**ክፍል V የተለያዩ የምግብ አይነቶችን በ24 ሰዓት ውስጥ ተመግበው ይሆን?**			
120	ከበቆሎ ፣ ከሩዝ ፣ ከስንዴ ፣ ከማሽላ፣ ከቴፍ፣ከዳጉሳ እና ማንናውም ከእነዚህ የሚዘጋጅ የምገብ ዓይነት (ዳቦ ፣ ፓስታ፣ብስኩት፣መኮሮኒ. ገንፎ) እና እንጀራ፣ ስኳር ድንች፣ ቀይ ስር፣ገንፎ ፣ ቂጣ …ወዘተ በ24 ሰዓት ዉስጥ ተመግበዋል?	0 አዎ 1 የለም	
121	ባቄላ ፣ አተር ፣ ሽምብራ፣ ምስር ፣ጓያ ያለበትን ምግብ በ24 ሰዓት ዉስጥ ተመግበዋል?	0 አዎ 1 የለም	
122	ባለፉት 24 ሰዓታት ውስጥ ለውዝ ወይም ዘሮችን በልተዋል?	0 አዎ 1 የለም	
123	ወተት፣ አይብ፣ ዕርጎ ፣ ቅቤ፣ አሬራ ፣ ወይም ሌላ የወተት ተዋጽዖዎችን በ24 ሰዓት ዉስጥ ተመግበዋል?	0 አዎ 1 የለም	
124	የበሬ፣የፍየልና፣የበግ እና የዶሮ ስጋ፣ጉበት፣፣ኩላሊት፣አሳ በ24 ሰዓት ዉስጥ ተመግበዋል?	0 አዎ 1 የለም	
125	የዶሮ እንቁላል ፣ የዳክዬ ፣ የቆቅ ፣ የጅግራ …ወዘተ እንቁላል በ24 ሰዓት ዉስጥ ተመግበዋል?	0 አዎ 1 የለም	
126	ጥቅል ጎመን፣ካሳቫ፣ሰላጣ፣ የአበሻ ጎመን በ24 ሰዓት ዉስጥ ተመግበዋል?	0 አዎ 1 የለም	
127	ዱባ፣ካሮት ፣የበሰል ፡ማንጎ፣የበሰለ ፓፓያ፣አናናስ፣ሎሚ፣እንጆሪ፣ ኮክ እና ከእነዝህ የተሰሩ ጭማቂዎች በ24 ሰዓት ዉስጥ ተመግበዋል?	0 አዎ 1 የለም	
128	ቲማቲም ፣ ሽንኩርት ፣ ቃሪየ፣ የባቄላ እሸት፣ የአተር እሸት እና የዱር ቅጠላቅጠሎች በ24 ሰዓት ዉስጥ ተመግበዋል?	0 አዎ 1 የለም	
129	ሙዝ፣ብርቱካን፣አቮካዶ እና ሌሎች ከእነዚህ የተሰሩ 100% የፍራፍሬ ጭማቂዎች በ24 ሰዓት ዉስጥ ተመግበዋል?	0 አዎ 1 የለም	
130	በእርግዝና ወቅት የአመጋገብ ልዩነትና አስፈላጊነትን በተመለከተ የምክር አገልግሎት አግተዋለ?	0 አዎ 1 የለም	
